# Effects of High-Frequency Electrical Stunning Current Intensities on Pre-Slaughter Stunning Stress and Meat Lipid Oxidation in Geese

**DOI:** 10.3390/ani11082376

**Published:** 2021-08-12

**Authors:** Lei Xu, Haiming Yang, Xiaoli Wan, Xin Zhang, Zhi Yang, Zhiyue Wang

**Affiliations:** 1 College of Animal Science and Technology, Yangzhou University, Yangzhou 225009, China; xlei@yzu.edu.cn (L.X.); hmyang@yzu.edu.cn (H.Y.); wanxl@yzu.edu.cn (X.W.); ZFF9962@163.com (X.Z.); 2 Joint International Research Laboratory of Agriculture and Agri-Product Safety of Ministry of Education of China, Yangzhou University, Yangzhou 225009, China; zhiyang@yzu.edu.cn

**Keywords:** electrical stunning, goose, stunning stress, hypothalamus-pituitary-adrenal axis, DPPH·EA, malondialdehyde, adrenocorticotropic hormone, superoxide dismutase

## Abstract

**Simple Summary:**

China produces the largest number of meat-producing geese in the world. However, few studies have investigated the effects of electrical stunning parameters on stunning stress or meat lipid oxidation in geese. We aimed to evaluate the stunning stress and meat lipid oxidation in geese stunned at a high electrical frequency level with different electrical current intensities in a water bath. Stunning the geese at 40 mA resulted in the minimum stunning stress based on the gene expression and hormones released in the hypothalamus–pituitary–adrenal axis. Stunning geese with 40 mA resulted in low stunning stress and lipid oxidation in breast meat (d 2), with moderate antioxidant capacity in the meat of the breast (d 2) and thigh (d 0) as compared with 100 mA. A combination of 40 mA, 500 Hz, 10 s per bird is suitable for the electrical stunning of geese and for the alleviation of stunning stress and meat lipid oxidation. This study may help to alleviate stunning stress and improve meat quality in the geese production industry.

**Abstract:**

Intensive slaughtering with electrical stunning (ES) is replacing traditional manual slaughtering of geese in China. This study aimed to assess stunning stress and meat lipid oxidation in geese stunned by high-frequency current intensities. Forty male Yangzhou geese, 92 days old, were randomly allocated into five treatments with eight replicates per treatment. The geese in the control group were not stunned, while the other birds were stunned by alternating current (AC) in a water bath. Each bird received a current intensity of 20 mA (E20mA), 40 mA (E40mA), 70 mA (E70mA), or 100 mA (E100mA) for 10 s at 500 Hz. The gene expression of c-jun N-terminal kinase 1 tended to decrease in the E40mA birds (*p* = 0.08). Stunning with 40 mA resulted in the maximum serum uric acid and urea among the ES groups and decreased serum adrenocorticotropin and creatine kinase (*p* < 0.01) compared with 70 mA and 100 mA. Increasing the current intensity reduced the diphenylpicrylhydrazyl free radical elimination ability and total-superoxide dismutase linearly in goose breast meat at d 2 and in thigh meat at d 0 (all *p* < 0.01). Stunning geese with 40 mA at 500 Hz for 10 s could alleviate stunning stress and meat lipid oxidation.

## 1. Introduction

China is the largest producer and market for meat-producing geese in the world. China produces about 5.19 billion geese, accounting for approximately 3.2 million tons of meat in 2020 [1]. Meat produced by a goose is rich in unsaturated fatty acids [2], which are beneficial for human health but sensitive to lipid oxidation. If the production of reactive oxygen species cannot be eliminated or neutralized to a normal level, lipid oxidation occurs [3]. Lipid oxidation can change the color, protein structure, shelf life, smell and taste of meat [4,5,6,7]. Compared with duck and chicken species, geese may be the most sensitive to lipid peroxidation [8].

Electrical stunning (ES) is one factor that influences bleed-out efficiency and lipid oxidation in broilers [9,10,11] and geese products [12], as observed in our previous study. Pre-slaughter electrical stunning is the primary method used world-wide to humanely slaughter poultry and for its easy operation [13]. Electrical water-bath stunning accounts for about 74% of pre-slaughter stunning in broilers in the Chinese slaughter industry [14]. Lipid oxidation could be affected by ES variables [10,11] and CO_2_ concentrations [15] in chicken slaughtered after stunning. A high electrical frequency can improve fatty liver production in geese when combined with high current intensities [16]. A high frequency can also improve the meat quality in chickens [17]. High frequencies were observed to change the extent of lipid peroxidation in chicken meat [10]. Our previous study [12] demonstrated that geese stunned with 40 mA at 500 Hz had less lipid oxidation in the liver as compared with geese slaughtered without stunning. Scientists have studied the influence of ES on blood loss, meat quality, and liver quality in over-fed geese [16,18]. However, little research has been performed to evaluate the effect of ES parameters on lipid peroxidation in meat-producing geese. We hypothesized that the proper ES current intensity could reduce meat lipid oxidation in geese stunned at 500 Hz.

Pre-slaughter stunning could also affect the degree of stress and meat quality in poultry. High frequencies could improve meat quality, but whether it stimulates stress depends on the combined current intensity [17]. Many studies have been done to evaluate the humanity of various stunning methods on poultry. These studies involve the effect of CO_2_ stunning on animal welfare in broilers [19], low atmospheric pressure stunning (LAPS), N_2_, and CO_2_ stunning on broiler breeders [20], ES voltages and waveforms on turkeys [21], and captive bolt stunning, gas or electrical stun-to-kill methods on heavy turkeys [22]. These evaluations were based on the criteria of stress hormones [10,15,17,19,22], physical responses [20,21], and electroencephalograms (EEGs) [21,22], etc. Geese are sensitive to various environmental factors. However, few studies on the effects of ES current intensity on stunning stress in meat goose have been conducted. We speculated that the proper current intensity could reduce stunning stress in meat geese.

The present study aimed to evaluate the stunning stress and meat lipid oxidation in meat geese stunned at a high electrical frequency level with different electrical current intensities in a water bath. This study could help to alleviate stunning stress and improve meat quality in the geese production industry.

## 2. Materials and Methods

### 2.1. Geese and Environment

We selected 100 28-day-old male Yangzhou geese, with a bodyweight of 1200 ± 200 g for the experiment. Birds were raised on a plastic slat floor, with free access to feed and water. The feed was a corn-soybean diet formulated with a ME of 10.89 MJ/kg and crude protein at 16.60% [12]. When the birds were 92 days old, 40 geese with body weights of around 3.80 (±0.15) kg were distributed into 5 groups. Approval was acquired from the Animal Care and Use Committee at the Yangzhou University (NO. YZUDWSY 2017-09-06).

### 2.2. Electrical Stunning System

The stunning system and stunning process were done according to [12]. A current and frequency transformer (SQ series, Chang Xun Machinery, Nanjing, China) was connected to a sinusoidal alternating current (AC, 220 V, 50 Hz) to produce designed electricity. A single goose was treated for each stunning time to avoid the uneven distribution of the current intensity. The shackle and electrical bar were tightly fixed to avoid uneven electrical conduction. The stunning duration was set at 10 s.

### 2.3. Grouping, Stunning Parameters, and Slaughter

Geese were randomly grouped into five treatments. Each treatment had eight replicates and each replicate had one goose. Geese in the control group were not stunned. They were hung upside down in the slaughter line, and sacrificed by bleeding via cutting the carotid artery and jugular vein on both sides of the neck to simulate the traditional slaughter method in live bird market. The other birds were bled after ES for 10 s with AC at 500 Hz in the water bath. The birds received a current intensity of 20 mA (E20mA), 40 mA (E40mA), 70 mA (E70mA), or 100 mA (E100mA) according to a previous study [12]. The bleeding of the ES groups was performed in the same way as the control group.

### 2.4. Sampling

The sampling devices were sterilized. Blood was sampled in procoagulant tubes during slaughter, then centrifuged for 10 min at 2500 r/min. The whole hypothalamus and adrenal gland were harvested into enzyme-free tubes, immediately frozen in liquid nitrogen, and transferred to −80 °C for measurement of gene expression. Two pieces of meat (5 g) were taken from the first layer of breast (*Pectoralis Major*) and thigh (*Musculus Iliofibularis*) muscles for detection of malondialdehyde (MDA), and diphenylpicrylhydrazyl free radical elimination ability (DPPH·EA), and total superoxide dismutase (T-SOD). One piece of muscle was stored at −20 °C after sampling (d 0); another piece was kept at 4 °C for 48 h (d 2) and then stored at −20 °C. All the samples were measured within six months. Samples from all the treatments were measured in the same batch for each variable.

### 2.5. Serum Variables

Uric acid (UA), urea, and creatine kinase (CK) were measured via the enzyme colorimetry method, urine enzyme continuous monitoring method, and continuous detection method, respectively, according to the instructions in the commercial kits. The UA, urea, and CK kits were bought from Shanghai Rongsheng Biopharmaceutical Co., Ltd. (Shanghai, China), and measurements were performed on an automatic biochemical analyzer (AU 2700, Olympus, Tokyo, Japan). Adrenocorticotropic hormone (ACTH) was determined via iodine [^125^I] ACTH radioimmunoassay kit. Cortisol was measured with an iodine [^125^I] cortisol radioimmunoassay kit. Both the kits were bought from the Beijing North Institute of Biotechnology Co., Ltd. (Beijing, China). The ACTH and cortisol were measured with GC-911γ gamma radioimmunoassay counter produced from USTC Chuangxin Corporation Limited Zonkia Branch (Hefei, China).

### 2.6. Lipid Oxidation and Antioxidant Capacity in Meat

The reaction between lipid peroxidation products and thiobarbituric acid (TBA) leads to MDA, which turns into pink MDA-TBA2 adducts (TBARS) [23]. Frozen meat (0.50 g) was cut off and homogenized with 4.50 mL saline in a sterile tube on ice and centrifuged at 2500 r/min at 4 °C for 10 min to get a 10% stock solution. The stock solution was divided into four tubes and stored at −20 °C. The T-SOD, MDA and total protein was determined with the hydroxylamine method (T-SOD kit, A001-3), TBARS method (MDA kit, A003-1), and Coomassie brilliant blue method (protein kit, A045-2), respectively, according to the instructions of the manufacturer. The DPPH·EA was measured using a diphenyl-1-picrylhydrazyl free radical (DPPH·) kit (A153-1-1) and the results were compared with a standard curve obtained with Trolox (TE), according to our previous study [12]. Means were shown as μmol Trolox equivalent (TE)/g prot. All the kits were produced by Nanjing Jiancheng Bioengineering Institute (Nanjing, China) [12]. The optical density of the termination solution was measured with a Multiskan FC microplate reader produced by Thermo (Waltham, MA, USA). The results of SOD and MDA at d 0 and d 2 postmortem were recorded as T-SOD_d0_, T-SOD_d2_, MDA_d0_, MDA_d2_, respectively.

### 2.7. Isolation of Total RNA

We selected control, E20mA and E100mA for further analysis of gene expression, based on the results of the serum variables. The mRNA expression was measured using real-time PCR using green fluorescent protein (GFP) as external control and β-actin as a housekeeping gene. Samples were determined for the mRNA expressions of c-jun N-terminal kinase 1 (JNK1, also known as mitogen-activated protein kinase 8, MAPK8), caveolin 1 (CAV1), and melanocortin two receptors (MC2R), GFP, and beta-actin (β-actin). Total RNA from an 80 mg meat sample was extracted in 1000 μL TRIzol Reagent produced by Invitrogen (Frederick, MD, USA) added with 25 fmol synthetic constructs of green fluorescent protein (GFP) RNA (CR100-01, Tiangen Biotech Co., Ltd., Beijing, China). The RNA quality and RNA concentration were evaluated according to a previous study [15].

### 2.8. Reverse Transcription and cDNA Synthesis

The cDNA was synthesized with a reverse transcription kit (Thermo, Shanghai, China). Briefly, 1 μg total RNA was reacted with 1 μL random primer in a 12 μL reaction system at 65 °C for 5 min. Then the mixture was combined with 4 μL 5 × Buffer, 2 μL dNTP Mix, 1 μL Protector RNase Inhibitor, and 1 μL transcriptase, and gently mixed to make a 20 μL reaction system. The reverse transcription PCR was performed with negative control (without transcriptase). The program was set as follows: 5 min at 25 °C, followed by 60 min at 42 °C, then 5 min at 70 °C. Finally, cDNA was obtained and stored at −20 °C for the following PCR amplification. The purity of cDNA was checked via gel electrophoresis.

### 2.9. Real-Time Quantitative PCR

The mRNA was quantified in an iCycler IQ multi-color (Bio-Rad Laboratories, Hercules, CA, USA). A 10 μL reacting system was provided with one μL c DNA, 0.3 μL sense primer and antisense primer (10 μM), 5.0 μL 2× Master Mix (Roche, Shanghai, China), and 3.4 μL double-distilled water. A sealing film was carefully placed on the PCR plate and centrifuged for a short time. The PCR plate was put on ice before setting the real-time PCR program. The reaction included one cycle at 95 °C (10 min), 45 cycles at 95 °C (15 s), and one cycle at 60 °C (60 s). Fluorescence was collected for each cycle to make a melting curve. The temperature system was 95 °C, 10 s followed by 60 °C, 60 s, and then 95 °C, 15 s. The fluorescence was collected from 60 °C to 99 °C (+0.05 °C/s).

Other information on the PCR is shown in Table 1. Measurements were conducted in triplicate, and the average cycle threshold (CT) values were taken as the final value. The average CT values of GFP and β-actin were used to normalize the relative mRNA level of target genes using the 2^−ΔΔCT^ method [24].

### 2.10. Statistical Analysis

Data were analyzed via the software of SPSS vs. 20.0 (SPSS Inc., Chicago, IL, USA). Homogeneity analysis was performed to test whether the data followed the normal distribution. If the data followed a normal distribution, one-way Anova was conducted. Then Duncan’s multiple range tests were done to compare the significant differences among groups. Otherwise, data were analyzed by Welch’s Anova. Comparisons of the MDA between tissues or between storage times were performed via paired-sample T-tests. Results are shown as means with SEM. A *p* < 0.05 indicates a significant difference.

## 3. Results

### 3.1. Serum Variables

The impacts of electrical current intensities on serum variables in geese are displayed in Table 2. Compared with E40mA and the control, stunning geese with E70mA and E100mA increased serum ACTH (*p* < 0.01) and CK (*p* < 0.01) levels; stunning with E70mA decreased urea (*p* = 0.05); and E20mA reduced the UA (*p* = 0.06) content. The serum variables did not differ (*p* > 0.05) between E40mA and the control group. The concentration of serum cortisol was not affected (*p* > 0.05) by stunning methods.

### 3.2. Lipid Oxidation

The MDA in the meat represents lipid oxidation. The effects of electrical stunning (ES) methods on MDA in goose meat are shown in Table 3. Stunning with E40mA resulted in the lowest MDA (*p* < 0.01) content in breast meat among all the treatments. However, the MDA content in both breast meat and thigh meat was not affected (*p* > 0.05) by stunning on the day of slaughter or in the thigh meat at two days (*p* > 0.05) postmortem.

**Figure 1:**
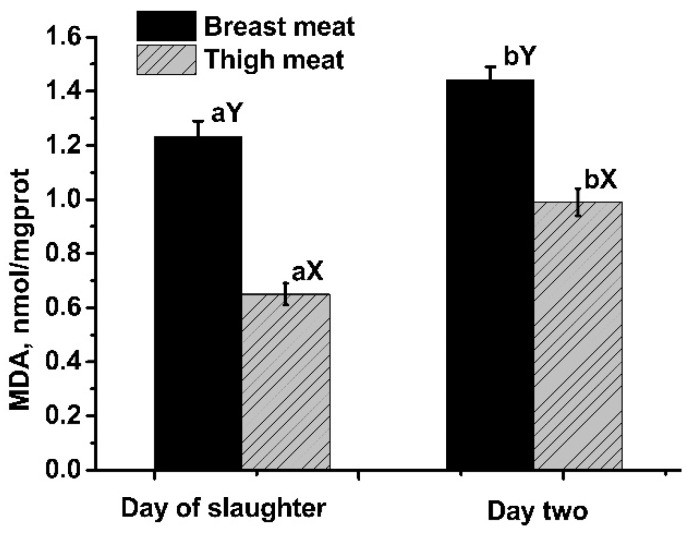
Effects of tissue and storage time on malondialdehyde (MDA) concentration. Means with no common superscripts between storage times (a, b) or tissues (X, Y) differ significantly (all *p* < 0.01).

The tissue and storage time affected MDA concentration in geese (Figure 1). Breast meat had a higher (*n* = 40, both *p* < 0.01) MDA level than thigh meat at d 0 and d 2. Storing meat for two days elevated (*n* = 40, both *p* < 0.01) MDA content in both the breast and thigh.

### 3.3. DPPH·EA and T-SOD Activity

The effect of ES methods on DPPH·EA and T-SOD activity in goose meat are shown in Table 4. Stunning geese with increasing current intensity linearly reduced DPPH· elimination ability, the enzyme activity of T-SOD_d2_ in breast meat and T-SOD_d0_ in the thigh meat of geese (all *p* < 0.01). Stunning geese with 20 mA resulted in higher (*p* = 0.02) DPPH· EA in thigh meat, higher (*p* < 0.01) T-SOD_d2_ in breast meat and higher (*p* = 0.05) T-SOD_d0_ in thigh meat compared with 100 mA.

**Figure 2:**
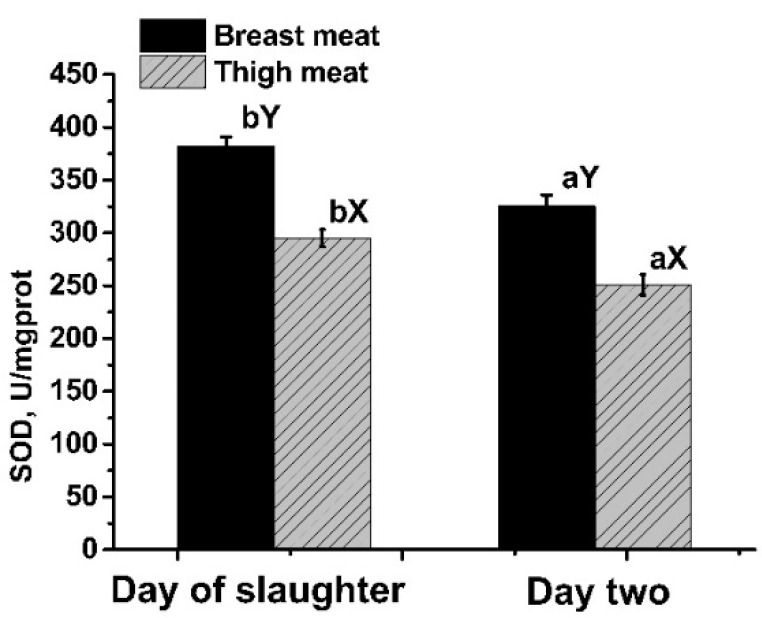
Effects of tissue and storage time on total-superoxide dismutase (T-SOD) activity. Means with no common superscripts between storage times (a, b) or tissues (X, Y) differ significantly (all *p* < 0.01).

The tissue and storage time affected T-SOD activity (Figure 2). Breast meat had a higher (*n* = 40, both *p* < 0.01) T-SOD level than thigh meat both at d 0 and d 2. Storing meat for two days reduced (*n* = 40, both *p* < 0.01) T-SOD activity in both the breast and thigh.

### 3.4. Gene Expressions in Hypothalamus and Adrenal Gland

The JNK1 mRNA expression in the hypothalamus slightly decreased (*p* = 0.08) by E40mA compared with the control (Table 5). Stunning with E40mA or E100mA did not affect (*p* > 0.05) CAV1 or MC2R gene expression in the adrenal gland. Stunning with E100mA resulted in the same (*p* > 0.05) JNK1, CAV1, and MC2R level as the control.

## 4. Discussion

### 4.1. Stunning Stress

The hypothalamic–pituitary–adrenal axis is a classical pathway that responds appropriately to stressful events [25]. The hypothalamus excretes corticotropin-releasing hormone, which stimulates the excretion of ACTH in the anterior pituitary gland. When ACTH is circulated to the adrenal cortex, it transduces signals to stimulate the synthesis of glucocorticoids and mineralocorticoids [26,27,28], e.g., corticosterone and cortisol. The responsiveness of the adrenals to ACTH was suggested as a useful way to monitor a bird’s reaction to stressful situations [29]. Melanocortin-2 receptor (MC2R), a coupled member of the melanocortin receptor family, is an ACTH receptor [30]. Production of ACTH-induced hormone is mediated by the pathway involving the participation of MC2R- cyclic adenosine monophosphate in adrenocortical cells [31]. Overexpression and spatiotemporal regulation of CAV1 is involved in the mediation of oxidative stress [32]. In our study, the mRNA expression of CAV1 and MC2R was not affected by ES current intensities in the adrenal gland. This may explain why the serum cortisol was not affected by the electrical current intensities from 20 to 100 mA at an electrical current frequency of 500 Hz. The minimum ACTH in the 40 mA group indicated the minimum stunning stress in geese, whereas stunning geese with 70 mA and 100 mA may cause higher electrical stunning stress based on more elevated serum ACTH levels. Different electrical stunning and gas stunning parameters could alter plasma cortisol or corticosterone in poultry [10,14,15,17,19,22,33]. Monitoring the response of serum ACTH to stunning electrical stress in geese was more sensitive than monitoring the concentration of serum cortisol in the present study.

A high CK concentration in the blood is one of the stress indicators [34]. The effect of stunning stress on serum CK in geese has rarely been reported. High loading density increased the CK concentration in blood as compared to lower loading density during pre-slaughter transport of pigs [35]. Heat stress increased the CK level, which could be reduced by purified rosemary extract in chickens [34]. In the present study, a higher CK level was found in groups E70mA and E100mA than that in E20mA and E40mA, suggesting that a current intensity more than 70 mA may stimulate stunning stress in geese.

The JNKs (members of the MAPKs family) are stress-response molecules that are activated via dual phosphorylation by the MAPK4 and MAPK7 with the JNK-interacting scaffold protein [36]. The JNKs signaling pathways are involved in the regulation of hypothalamic stress induced by overnutrition [37]. Oxidative stress could activate protein kinase-JNK signaling pathways in liver cells [38]. Oxidative stress and stunning stress were observed to be associated with increased JNK1 and JNK2 gene expressions in breast muscle [10] and thigh muscle [11] of broilers stunned with different current intensities frequencies. The mRNA of JNK1 and JNK2 was down-regulated in breast muscle by gas stunning of broilers using 40% and 79% CO_2_ with 21% O_2_ as compared with broilers without stunning [15]. The appearance of epileptiform activity in birds’ brains is a requirement for ES according to the European Union Regulation of 1099/2009 [39]. The JNK activation triggers a neuroinflammatory response with synapses loss, cognitive impairment, neuronal cell death, and epilepsy [40]. In Europe, a minimum of 130 mA/(<200 Hz)/4 s is required by legislation to stun geese in a water bath to induce sudden unconsciousness in geese [39]. In China, low current ES is allowed in the poultry industry. In our study, the tendency of lower JNK1 mRNA level in the group of 40 mA may suggest less impairment of the hypothalamus and lower stunning stress in geese. The data for the three selected genes did not show a strong relationship with stunning stress. In the future, more sensitive genes may be screened through 16S RNA sequencing. The relationship between stress-induced gene expression and electroencephalograms (EEGs) in the brain also needs to be studied in the future to provide a better evaluation of geese welfare on the slaughter line. Taken together, stunning geese at 40 mA with 500 Hz may alleviate stunning stress in geese weighing around 3.8 kg.

### 4.2. Lipid Oxidation

Lipid oxidation is a vital factor that influences the nutritional value, shelf life, sensory quality, and safety of animal products [4,5,6,7]. Volatile compounds produced from lipid oxidation in goose products [6,7] could affect consumers’ desire to purchase. One of the significant biomarkers of lipid oxidation and oxidative stress is MDA [5]. Breast meat has a higher level of MDA consistent with the report in [41]. The reason might be that breast meat has a higher polyunsaturated fatty acids (PUFA) content than thigh meat in goose [42]. A report [43] showed that the redness values in breast muscle is the same or higher than the redness in thigh meat, indicating a high number of red fibers in the breast of Yangzhou geese. This may be another reason why the MDA level is higher in breast muscle. Based on our knowledge, no studies have been done to study the ES effects on meat lipid oxidation in geese. Stunning with E40mA resulted in the lowest MDA content in breast meat among all the treatments. Similarly, the MDA levels in geese livers were lower in the group with 70 mA, 500 Hz as compared with the group with 100 mA, 500 Hz on d 0 in a previous study [12]. Although lipid oxidation was not affected by ES methods at d 0, its level increased after more than two days storage time in both breast and thigh meat. Consistent with this study, we observed that MDA level was not affected in breast muscle by CO_2_ stunning concentrations at d 0 [15], or by ES methods [10] in broilers. As storage time was prolonged, MDA concentration was increased by the combination of high-frequency and low-current at d 1 and d 3 [10]. Similarly, stunning broilers with 40% CO_2_ resulted in higher MDA as compared with 79% CO_2_ at three days postmortem [15]. In the present study, 40 mA alleviated lipid oxidation compared to the control group, whereas other ES treatments did not affect lipid oxidation as compared with the control group. Some ES parameters could also reduce lipid oxidation, e.g., 130 mA, 60 Hz reduced MDA production in breast meat in broilers on d 1 and d 9 compared with the no-stunning group [10]. Parameters of 47 mA, 400 Hz and 86 mA, 1000 Hz reduced MDA levels in breast meat at 45 min postmortem as compared with no-stunning treatment [9]. Reasons for the inconsistency may be due to different ES variables (current intensities, frequency, duration), measured tissues (muscle or liver), body resistance (broilers or geese), and storage time. The present study’s results suggested that stunning with 40 mA, 500 Hz, 10 s may be used to protect meat lipid oxidation stability in geese. Note that only one bird was stunned in the water bath each time in our study, and total current intensity should be calculated according to the specific circuit if multiple birds are stunned simultaneously in a water bath.

### 4.3. Antioxidant Capacity

Serum UA, urea, and serum ammonia are primary metabolites of amino acids in birds [42]. An increase in UA and urea could impair renal function in birds [44]. However, under acute oxidative stress, UA may be a critical non-enzyme antioxidant that reduces reactive oxygen species to allantoin [45]. There are few studies on the effect of pre-slaughter events on serum UA and urea in geese. The ES group with 40 mA had the highest level of both UA and urea among the ES treatments, indicating that stunning with 40 mA may activate amino metabolism to increase the potential for higher antioxidant status. Consistent with high UA and urea levels, a low level of MDA was observed in geese stunned with 40 mA. Similarly, serum uric acid was increased in broilers stunned with 47 mA and 400 Hz compared to broilers stunned with 67 mA and 160 Hz [46]. Uric acid was higher with 86 mA than 130 mA in broilers in our previous study [10]. The elevation of uric acid and urea nitrogen in blood suggests the mobilization of protein to fight the stress caused in broilers stunned with 47 mA and 400 Hz in study performed by [46]. Gas stunning with 40% CO_2_ did not affect UA as compared with 60% CO_2_ [46] and 80% CO_2_ [15]. In the present study, the relatively high level of UA and urea in geese stunned with 40 mA may partly contribute to a lower level of lipid oxidation in geese meat.

The superoxide dismutases (SODs, three isoforms) form a critical antioxidant defense line against superoxide anion radicals and reactive oxygen species [47], which help to keep a balance in oxidation reduction. In the present study, MDA content was higher in breast meat than that in thigh meat. Consistently, T-SOD activity was higher in goose breast meat than in thigh meat, suggesting that T-SOD is a group of vital enzymes that prevent breast meat in geese from oxidative stress and lipid oxidation. Stunning geese with increasing current intensity linearly reduced DPPH· elimination ability, T-SOD_d2_ in breast meat and T-SOD_d0_ in thigh meat of geese, with a consistently low antioxidant capacity in the meat of the 100 mA group. Similarly, DPPH· elimination ability was reduced in high current intensity compared with low current intensity in goose liver [12]. The lowest T-SOD was consistently observed in the liver of geese stunned with 100 mA in our previous study [12]. In another study by our team, mRNA of SOD_2_ was slightly upregulated by the treatment with lower current intensity, but the activity of SOD was not affected in breast meat within 24 h [10]. The enzyme activity of SOD_d0_ and SOD_d1_ was slightly increased with 130 mA, 60 Hz in the thigh meat of broilers compared with no stunning [11]. Using 79% CO_2_ stunning enhanced SOD_d1_ activity in both breast and thigh meat compared with the no stunning group [15]. The stimulation of T-SOD activities in muscle was different in the breast and thigh at d 0 and d 2 in the present study. Similarly, antioxidant status was not a synchronous change in the two kinds of meat [10,11,15]. The SOD activity in thigh meat increased one day after ES [11]. As the storage time of breast and thigh meat increased to two days, the T-SOD activity decreased, resulting in a rise in lipid oxidation in geese. Although the maximum DPPH· elimination ability and SOD activity was not in E40mA, the UA and urea may work together to maintain lipid oxidation at a low level in geese stunned with 40 mA. The reason might be that lipid oxidation is controlled by multiple antioxidant enzymes, antioxidant proteins, and non-enzyme antioxidants that react differently to an ES parameter [10,11,15].

## 5. Conclusions

The use of 40 mA at 500 Hz alleviated the stunning stress in geese as reflected by lower serum ACTH, serum CK and a lower mRNA level of JNK1 in the hypothalamus. Increasing current intensity reduced the DPPH· elimination ability and SOD activity in goose meat. The combination of 40 mA with 500 Hz enhanced the antioxidant ability and reduced the lipid oxidation as reflected by lower MDA content in meat, higher serum UA, more elevated urea, moderate SOD activity and DPPH· elimination ability in meat. Parameters of 40 mA and 500 Hz are appropriate to stun geese that weigh around 3.8 kg and result in low stunning stress and meat lipid oxidation.

## Figures and Tables

**Table 1:** Primer information for PCR.

RNA ^1^	Sense	Antisense	Annealing (°C)	Product Size (bp)	GenBank Number
JNK1 (MAPK8)	ACAGGGGATAGTATGTGCAGCTT	ATGGTCGGCTTAGCTTCTTGAT	60 °C	74	XM_013198744.1
CAV1	CAACGACGACGTGGTCAAGA	AGAGCCATAGGAATGCCAAAGA	60 °C	162	XM_013190822.1
MC2R	GACCTTCCAATCTAATCAAACACCT	GATGACAGCAATAAGGACAAGCA	60 °C	176	XM_013179521.1
β-actin	CAACGAGCGGTTCAGGTGT	ATGATGGAGTTGAAGGTGGTCT	60 °C	96	XM_013174886.1
GFP	TTGAAGATGGAAGCGTTCAACTAGC	AGTTCATCCATGCCATGTGTAATCC	60 °C	196	*

^1^ JNK1, c-jun N-terminal kinase 1, also known as mitogen-activated protein kinase 8 (MAPK8). CAV1, caveolin 1; MC2R, melanocortin 2 receptor; β-actin, beta-actin; GFP, green fluorescent protein. * GFP was a commercial synthetic construct of short strand RNA.

**Table 2:** Effects of electrical current intensities on serum variables in geese.

Items ^1^	Stunning Methods ^2^					SEM	*p* Values
	Control	E20mA	E40mA	E70mA	E100mA		
ACTH, pb/mL	27.10 ^ab^	28.02 ^ab^	24.20 ^a^	32.67 ^bc^	34.57 ^c^	1.01	<0.01
Cortisol, ng/mL	20.49	19.60	19.31	18.48	20.35	0.49	0.72
CK, U/L	1347 ^a^	1741 ^a^	2081 ^ab^	3640 ^c^	2805 ^bc^	193.6	<0.01
Urea, mmol/L	1.08 ^b^	0.82 ^ab^	0.90 ^b^	0.70 ^a^	0.88 ^ab^	0.04	0.05
UA, umol/L	388.0	269.0	379.5	324.2	378.6	15.12	0.06

^1^ ACTH, adrenocorticotropic hormone; CK, creatine kinase; UA, uric acid. ^2^ Geese were not stunned in the control group; the other birds were stunned in a water bath with an alternative current intensity of 20 mA (E20mA), 40 mA (E40mA), 70 mA (E70mA), or 100 mA (E100mA), and a stunning period of 10 s at 500 Hz. ^a–c^ Means within a row without the same superscripts differ significantly (*p* < 0.05).

**Table 3:** Effects of electrical current intensities on malondialdehyde (MDA) in goose meat at 0 to 2 days postmortem.

Items	Stunning Method ^1^					SEM	*p* Values
	Control	E20mA	E40mA	E70mA	E100mA		
Breast meat							
MDA_d0_, nmol/mgprot	1.52	1.16	1.16	1.16	1.12	0.06	0.22
MDA_d2_, nmol/mgprot	1.62 ^b^	1.50 ^b^	1.11 ^a^	1.51 ^b^	1.50 ^b^	0.05	< 0.01
Thigh meat							
MDA_d0_, nmol/mgprot	0.83	0.52	0.60	0.67	0.64	0.04	0.17
MDA_d2_, nmol/mgprot	1.22	0.99	0.87	1.01	0.86	0.05	0.08

^1^ Geese were not stunned in the control group; the other birds were stunned in a water bath with an alternative current intensity of 20 mA (E20mA), 40 mA (E40mA), 70 mA (E70mA), or 100 mA (E100mA), and a stunning period of 10 s at 500 Hz. ^a,b^ Means within a row without the same superscripts differ significantly (*p* < 0.05).

**Table 4:** Effect of electrical current intensities on DPPH·EA and T-SOD activity in goose meat at 0 to 2 days postmortem.

Items ^1^	Stunning Method ^2^					SEM	*p* Values	
	Control	E20mA	E40mA	E70mA	E100mA		Groups	Linear
Breast meat								
DPPH·EA, μmolTE/g prot	0.19	0.18	0.19	0.19	0.19	<0.01	0.20	0.49
T-SOD_d0_, U/mgprot	389	371.3	403.3	375.8	371.3	8.49	0.74	0.62
T-SOD_d2_, U/mgprot	360.2 ^b^	352.9 ^b^	338.4 ^ab^	298.2 ^ab^	277.4 ^a^	10.62	<0.01	<0.01
Thigh meat								
DPPH·EA, μmolTE/g prot	0.21 ^b^	0.21 ^b^	0.19 ^a^	0.19 ^a^	0.19 ^a^	<0.01	0.02	<0.01
T-SOD_d0_, U/mgprot	360.22 ^b^	352.94 ^b^	338.35 ^ab^	298.16 ^ab^	277.43 ^a^	10.62	0.05	<0.01
T-SOD_d2_, U/mgprot	280.1	266.1	221.2	251.2	236.4	10.03	0.38	0.18

^1^ DPPH·EA, diphenylpicrylhydrazyl free radical (DPPH·) elimination ability; TE, Trolox equivalent. T-SOD, total-superoxide dismutase. ^2^ Geese were not stunned in the control group; the other birds were stunned in a water bath with an alternative current intensity of 20 mA (E20mA), 40 mA (E40mA), 70 mA (E70mA), or 100 mA (E100mA), and a stunning period of 10 s at 500 Hz. ^a,b^ Means within a row without the same superscripts differ significantly (*p* < 0.05).

**Table 5:** Effect of electrical stunning (ES) methods on gene expression in the hypothalamus and pituitary.

Items ^1^	Stunning Methods ^2^			SEM	*p* Values
	Control	E40mA	E100mA		
JNK1	1.00	0.70	0.90	0.06	0.08
CAV1	1.07	0.87	0.81	0.09	0.45
MC2R	0.93	0.76	0.70	0.05	0.17

^1^ JNK1, CAV1, MC2R represent c-jun N-terminal kinase 1 (also called mitogen-activated protein kinase 8, MAPK8) from the hypothalamus, caveolin 1, and melanocortin 2 receptor from the adrenal gland, respectively. ^2^ Geese were not stunned in the control group; the other birds were stunned in a water bath with an alternative current intensity of 20 mA (E20mA), 40 mA (E40mA), 70 mA (E70mA), or 100 mA (E100mA), and a stunning period of 10 s at 500 Hz.

## Data Availability

The data in this study are available on reasonable request from the corresponding author.
